# Acute Cholangitis following Biliary Obstruction after Duodenal OTSC Placement in a Case of Large Chronic Duodenocutaneous Fistula

**DOI:** 10.1155/2015/647806

**Published:** 2015-06-21

**Authors:** Yaseen Alastal, Tariq A. Hammad, Mohamad Nawras, Basmah W. Khalil, Osama Alaradi, Ali Nawras

**Affiliations:** ^1^Department of Internal Medicine, University of Toledo Medical Center, Toledo, OH 43614, USA; ^2^Department of Gastroenterology and Hepatology, University of Toledo Medical Center, Toledo, OH 43614, USA

## Abstract

Over-the-Scope Clip system, also called “Bear Claw,” is a novel endoscopic modality used for closure of gastrointestinal defect with high efficacy and safety. We present a patient with history of eosinophilic gastroenteritis and multiple abdominal surgeries including Billroth II gastrectomy complicated by a large chronic duodenocutaneous fistula from a Billroth II afferent limb to the abdominal wall. Bear Claw clip was used for closure of this fistula. The patient developed acute cholangitis one day after placement of the Bear Claw clip. Acute cholangitis due to papillary obstruction is a potential complication of Bear Claw placement at the dome of the duodenal stump (afferent limb) in patient with Billroth II surgery due to its close proximity to the major papilla.

## 1. Introduction

Over-the-Scope Clip (OTSC) system, also called “Bear Claw,” is a novel endoscopic modality used for closure of gastrointestinal defect. Current retrospective studies suggest high efficacy and safety of the device. Herein we present a patient who developed acute cholangitis one day after placement of Bear Claw clip for closure of duodenocutaneous fistula from the afferent enteric limb of Billroth II. This complication is unreported in the literature after placement of Bear Claw clip.

## 2. Case Presentation

**Figure 1:**
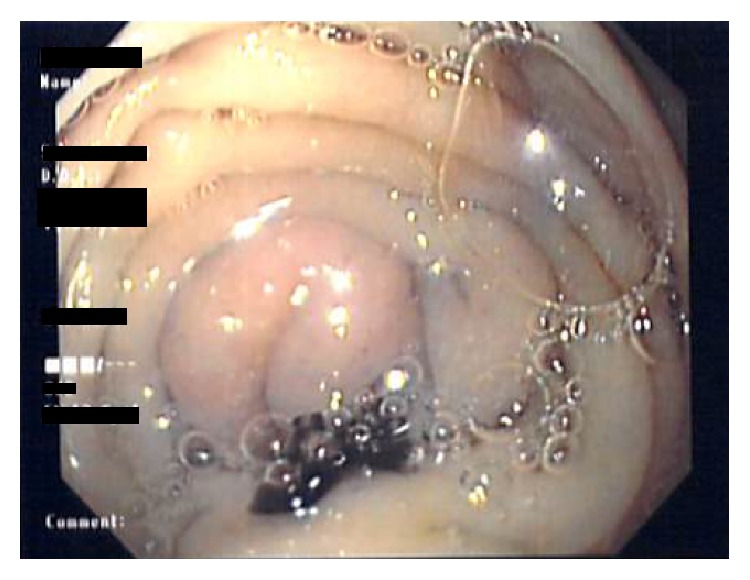
Spot draining from the internal fistula orifice at the dome of the blind limb of Billroth II after injecting it percutaneously.

**Figure 2:**
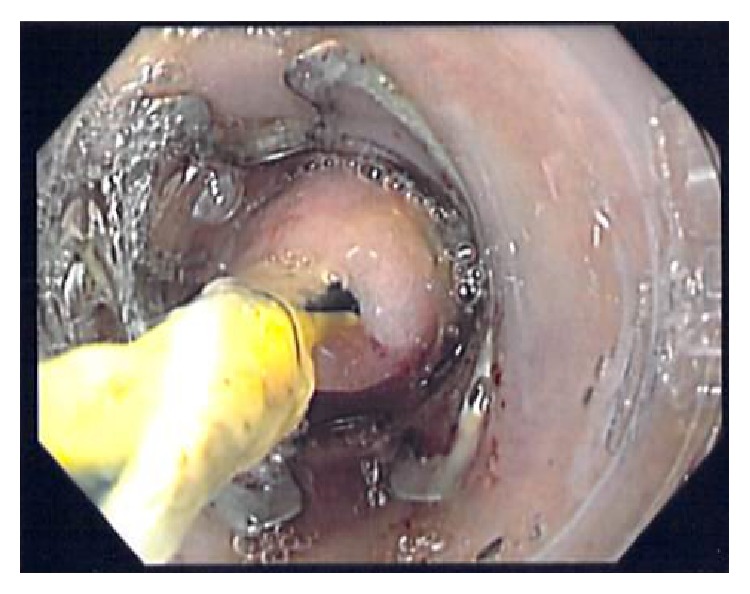
Enteric orifice of the fistula suctioned inside the cap of the Bear Claw with the guide wire still inside the fistula locating in the center.

**Figure 3:**
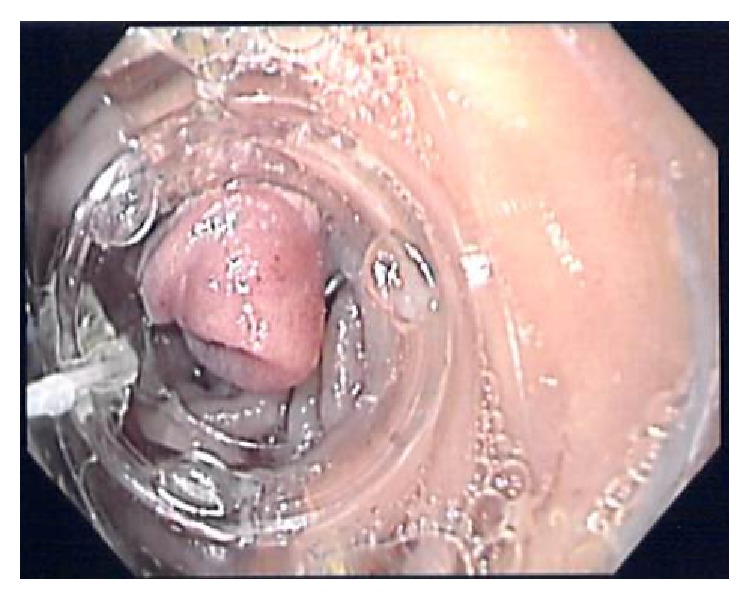
Bear Claw clip deployed at the enteric orifice of the fistula within the afferent limb.

**Figure 4:**
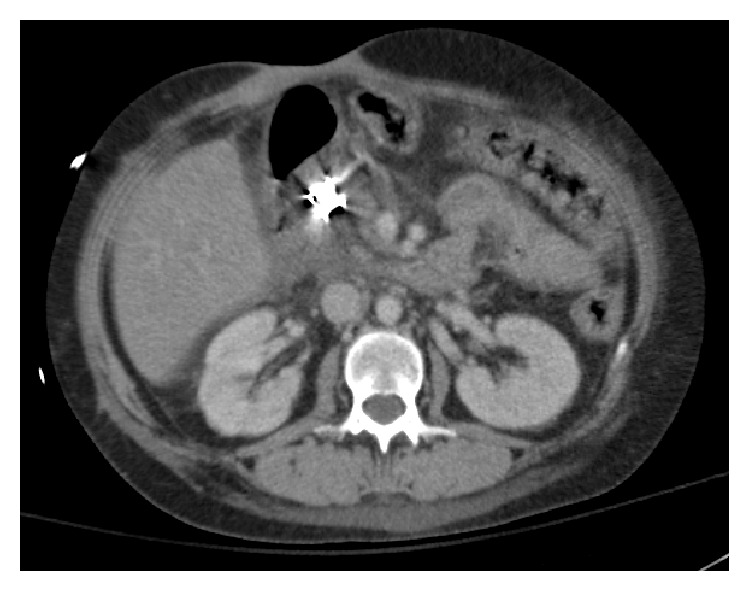
Bear Claw clip placed in the afferent loop of the Billroth II with mild edema surrounding this region.

**Figure 5:**
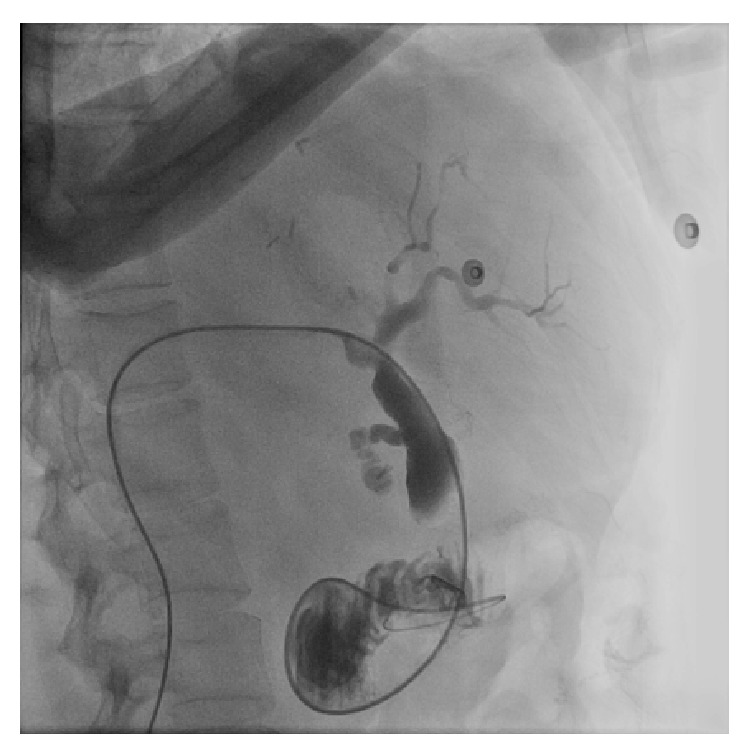
Percutaneous transhepatic cholangiography showed diffuse dilation of the biliary tree and the guide wire passing through the major papilla just adjacent to the Bear Claw clip.

28-year-old female patient with history of eosinophilic gastroenteritis and multiple abdominal surgeries including Billroth II gastrectomy referred to our hospital for treatment of duodenocutaneous fistula. She had large chronic duodenocutaneous fistula measuring about 0.8 cm in diameter extending from Billroth II afferent limb to the abdominal wall. Multiple previous attempts to close the fistula have failed. The patient was scheduled for elective cutaneous and enteric closure of the fistula with fibrin glue and Bear Claw endoclip placement, respectively. During the procedure, a pediatric colonoscope was introduced into the blind enteric limb; the major papilla was identified 3-4 cm proximal to the dome of the blind limb. India ink was injected through the cutaneous orifice of the fistula to localize the internal orifice in the dome of the blind limb (Figure 1). A 0.035 inch × 450 cm guide wire was introduced through the cutaneous orifice and advanced into the blind enteric limb under endoscopic and fluoroscopic guidance; the guide wire was grasped with rat tooth biopsy forceps. The pediatric colonoscope was then withdrawn with the guide wire. Subsequently, the scope was changed to an adult gastroscope. An 11/6 t Bear Claw endoclip was secured on the tip of the gastroscope; then the gastroscope with the Bear Claw endoclip was reintroduced over the guide wire into the afferent duodenal limb. The enteric orifice of the fistula was suctioned inside the cap of the Bear Claw with the wire still inside the fistula and scope channel (Figure 2). Once the fistula site at the dome of the afferent limb was seen filling the cap, the guide wire was removed and the clip was deployed successfully closing the enteric orifice of the fistula (Figure 3) (the attached video demonstrates the key portions of Bear Claw placement procedure in our patient). The position of the Bear Claw endoclip was confirmed endoscopically and fluoroscopically. Subsequently, the cutaneous orifice was identified and injected with fibrin glue. The patient tolerated the procedure well without immediate complications and was discharged home for outpatient follow-up. On the next day, the patient developed fever, jaundice, changes in mental status, and abdominal pain. Her vital signs were as follows: blood pressure: 124/68, pulse rate: 151 beats per minutes, temperature: 40.1°C, and respiratory rate: 28 per minute. Abdominal examination revealed diffuse tenderness. Laboratory work showed white blood cell counts of 5 × 10^9^/L (normal range: 4–10 × 10^9^/L), direct bilirubin of 3.3 mg/dL (normal range: 0–0.3 mg/dL), and Alkaline phosphatase, aspartate, and alanine aminotransferase levels of 255 U/L (normal range: 45–115 U/L), 272 U/L (normal range: 8–48 U/L), and 134 U/L (normal range: 7–55 U/L), respectively. Amylase and lipase were normal. Abdominal ultrasound revealed dilation of the common bile duct (1.2 cm) with intrahepatic biliary dilation. Abdominal CT scan showed mild edema surrounding the endoclip in the afferent loop of the Billroth II with no definite abscess or fluid collection (Figure 4). The pancreas was normal. HIDA scan suggested biliary obstruction. The patient was diagnosed with acute cholangitis secondary to biliary obstruction due to Bear Claw placement close to the major papilla grasping adjacent tissue. She was managed with empirical antibiotics and supportive care. Upon patient's family preference, percutaneous transhepatic cholangiography (PTC) was done rather than ERCP. PTC showed diffuse dilation of the biliary tree down to the papilla. Percutaneous transhepatic drainage was performed and the guide wire was advanced into the duodenum through the major papilla adjacent to the Bear Claw clip which was causing partial obstruction (Figure 5). An 8 French pigtail catheter was placed down to the duodenum. As a result, the patient had clinical and laboratory improvement. Five months later, the fistula did not close completely and the patient underwent laparotomy repair which involved dissection of the fistula from the surrounding adhesions and duodenotomy around it, followed by excision of the tract and the attached duodenal wall. The Bear Claw was not palpable on the duodenal wall during surgery which suggested spontaneous migration before surgery. The duodenal stump was sutured and covered with omental patch. The patient had an uneventful postoperative recovery with no recurrence of fistula after surgery.

## 3. Discussion

Therapeutic endoscopic procedures are rapidly evolving in the field of gastroenterology and have been showing promising results as minimal invasive interventions for treating gastrointestinal (GI) pathologies. Over-the-Scope Clip (OTSC) system is one of this novel endoscopic modalities which was approved in Europe in 2009 and then by the US Food and Drug Administration (FDA) in 2010 [1]. OTSC system has been used mainly in closure of luminal gastrointestinal defects like fistulas and perforation but other uses have been described like treatment of bleeding lesions, resection of submucosal neoplasms, and stents fixation [2]. When compared to standard endoclips, it showed more efficacy and better safety for closure of gastrostomies up to 18 mm in diameter [3]. This is because of its ability to grasp more tissues up to the entire thickness of the visceral wall and applying a greater compression force [1]. Overall success rates of OTSC in the literature range from 75% to 100% for closure of iatrogenic gastrointestinal perforations, 38% to 100% for closure of gastrointestinal fistulas, 50% to 100% for anastomotic leaks, and 71% to 100% for bleeding lesions [4]. According to an international multicenter retrospective study of 188 patients who underwent attempted OTSC placement for GI defects, the rate of successful closure of perforations (90%) and leaks (73.3%) was significantly higher than that of fistulae (42.9%) [5]. At this time, the safety of the device cannot be assessed accurately as most of published studies are case reports and retrospective studies, with no long term follow-up studies. However, most of the current case series did not report any complication after using the Bear Claw [3, 6–8]. A prospective multicenter study by Voermans et al. reported one patient who developed esophageal perforation while introducing the endoscope with the OTSC. They related that to the OTSC cap which has 2 mm protruding plastic rim that might make it slightly more traumatic than standard plastic caps widely used for rubber band ligation [9]. The same study also reported a patient with persistent perforation secondary to clip detachment after he underwent OTSC for colonic perforation after polypectomy; subsequently he died secondary to peritonitis [9]. Surace et al. reported one complication in their case series related to the delivery system of the clip as the anchor was blocked within the clip resulting in inability to be immediately withdrawn; then it was removed endoscopically after 7 days [10]. Acute cholangitis is a serious condition which has not been reported in the literature as a complication of this procedure. We describe the first case report of acute cholangitis developed directly after Bear Claw endoclip placement for closure of duodenocutaneous fistula in patient with Billroth II surgery. In our patient the enteric site of the fistula was at the dome of the blind enteric loop of Billroth II which is also the site of biliary tract drainage. The close proximity between the enteric site of the fistula and the major papilla within the afferent limb can explain the reason for developing this complication. Deploying a Bear Claw clip at the end of the afferent limb carries a risk of either complete obstruction of the major papilla by direct occlusion from the clip itself or partial obstruction by inducing edema at the surrounding tissue (as in our case). Deployment of Bear Claw clip in the duodenum has been described in the literature, as Haito-Chavez et al. included 11 cases in their retrospective analysis who underwent Bear Claw placement in the duodenum but there was no procedure related complication [5]. However none of the Bear Claw clips was reported to be deployed in the afferent limb (duodenal stump) of patient with Billroth II surgery.

Duodenocutaneous fistulae closure is considered a therapeutic challenge. With the internal orifice being at the afferent limb of Billroth II, this adds more difficulty into closing this fistula endoscopically. Only one case report in the literature described endoscopic closure of an iatrogenic duodenocutaneous fistula in a Billroth II afferent limb [11]. In this case report, endoscopist successfully used three standard endoclips to approximate the fistula opening which resulted from pinpoint duodenotomy defect occurring during dissection. Our reported case is the first case in the literature to use Bear Claw clip for closure of duodenocutaneous fistula from a Billroth II afferent limb. The reason for using Bear Claw endoclip instead of standard endoclip in our patient was related to the characteristics of the fistula. It was chronic, persistent despite multiple previous attempts of closure and relatively too large to be fully contained with the standard endoclip. In our case, the Bear Claw placement was unsuccessful in closing the fistula. To the best of our knowledge, clinical success rates of chronic fistula closure were poor compared to acute phase, and delayed closure remains challenging [12]. Additionally, the fistula leakage was large which decreased the chance of successful closure. Endoscopic vacuum therapy (EVT) is a new technique which was introduced in the last few years as an alternative treatment for esophageal perforation and some other postoperative leakages [13]. It involves placement of an endoscopic vacuum polyurethane sponge under direct endoscopic visualization followed by transnasal application of external vacuum. A very few successful treatments have been reported, adapting EVT by intraluminal approach to close duodenal leakage and guide biliary secretion [14, 15]. One report described an endoscopic pull-through technique where the polyurethane foam drainage was introduced through a visible percutaneous abdominal drainage site [16]. In our case, this technique could have been utilized to introduce the vacuum sponge onto the defect zone given the large defect size and high drainage output, which might help in healing the duodenocutaneous fistula.

We faced some technical challenges in performing this procedure. One of these challenges was advancing the gastroscope with the Bear Claw secured to the tip of the scope into the afferent limb. This was facilitated with wire guidance advanced percutaneously through the fistula and grasped with forceps and pulled into the scope channel. Another challenge was the mobility of the afferent limb which could have altered the suctioned position of the duodenal wall within the cap of the clip prior to deploying it despite the wire guidance. Moreover, despite using a guide wire to stabilize the suctioned portion of the duodenal stump to avoid the papilla, the endoclip was still deployed close to the papilla. Perhaps using the anchor might have stabilized the suctioned portion better than the guide wire. Also, Gentle care needs to be taken applying additional suction, especially when fistula is underlying nearby biliary tract. Endoscopist should be careful with placement of OTSC in the afferent limb of Billroth II surgery as it can directly or indirectly result in papillary obstruction and acute cholangitis.

## 4. Conclusion

Over-the-Scope Clip system is a new endoscopic modality with high efficacy and safety in closure of duodenocutaneous fistulas. Acute cholangitis due to papillary obstruction is a potential complication of OTSC placement at the dome of the duodenal stump (afferent limb) in patient with Billroth II surgery due to its close proximity to the major papilla.

## Supplementary Material

This video demonstrates the key portions of Bear Claw placement procedure in our patient. It starts with injection of India ink through the cutaneous orifice of the fistula to localize the internal orifice in the dome of the blind limb and identify it relation to the major papilla. Then it demonstrates the Bear Claw deployment process starting with suctioning the enteric orifice of the fistula inside the cap of the Bear Claw with the wire still inside the fistula. Once the fistula site at the dome of the afferent limb was seen filling the cap, the guide wire was removed and the clip was deployed. Last portion of the video demonstrates the PTC images which was done one day after the procedure and showed diffuse dilation of the biliary tree down to the papilla.
